# Contribution of Psychological Factors in Dropping out from Chronic Obstructive Pulmonary Disease Rehabilitation Programs

**DOI:** 10.1155/2014/401326

**Published:** 2014-02-13

**Authors:** Athanasios Tselebis, Epaminondas Kosmas, Dionisios Bratis, Argiro Pachi, Ioannis Ilias, Maria Harikiopoulou, Elpida Theodorakopoulou, Konstantinos Velentzas, Silvia Dumitru, Georgios Moussas, Nikolaos Siafakas, Nikolaos Tzanakis

**Affiliations:** ^1^Psychiatric Department, “Sotiria” General Hospital of Chest Disease, Mesogeion 152, 11527 Athens, Greece; ^2^Pulmonary Rehabilitation Centre, “Sotiria” General Hospital of Chest Disease, Mesogeion 152, 11527 Athens, Greece; ^3^Endocrinology Department, “Elena Venizelou” Hospital, 11521 Athens, Greece; ^4^Department of Thoracic Medicine, Medical School, University of Crete, 71 003 Heraklion, Greece; ^5^Laboratory of Epidemiology, Department of Social Medicine, Medical School, University of Crete, 71 003 Heraklion, Greece

## Abstract

Significant positive effects, particularly on psychological state in patients who completed the follow-up pulmonary rehabilitation programs, are indicated by a large number of studies. Yet, a remarkable proportion of selected patients drop out from these programs. In this study, we investigated existing differences on psychological variables among COPD patients who complete and those who drop out from pulmonary rehabilitation programs. The study included 144 patients, 43 (29.9%) of whom did not complete the program. SCL-90 was used for the assessment of psychological symptoms. On the SCL-90-R scale 55.6% of patients had abnormal findings. Patients who discontinued the program had higher rates of depression and somatization compared to those who completed it. Regarding the psychopathology scales of SCL-90R, we found that patients who discontinued the program showed higher levels of psychopathology on the scales of somatization, depression, paranoid ideation, and psychotism compared to those who completed the program. The final regression model showed that patients with low educational status and psychotism were more likely to leave the program. In conclusion, psychopathology contributes to patients dropping out from a COPD rehabilitation program; thus, psychological assessment prior to inclusion in rehabilitation programs may reduce dropouts.

## 1. Introduction

Pulmonary rehabilitation is defined as the evidence-based, multidisciplinary, and comprehensive intervention for patients with chronic obstructive pulmonary disease (COPD) who are symptomatic and often have reduced activity of daily living. Intervention incorporates individualized patient treatment, and aims to reduce symptoms, to optimize patients' functional status, to increase participation in treatment and to reduce health care costs through stabilization or improvement of systemic manifestations of disease [1, 2]. During the last decades pulmonary rehabilitation has emerged as a standard of care for patients with COPD and is included in guidelines and algorithms of care in patients with COPD [3, 4]. The positive effects in patients who completed follow-up in rehabilitation programs have been reported in many studies [5]; among those, a positive effect on the psychological state of patients is prominent [6, 7]. According to the literature COPD is clearly associated with high levels of psychological morbidity and the condition's objective severity alone is insufficient to predict clinical outcomes. Such levels of psychological morbidity detrimentally affect quality of life [8] in these patients (emotional and social role functioning and activities of daily livings and recreational pastimes [9]). In rehabilitation [10], health-related quality of life (HRQL) measures, such as disease-specific health status (St. George's respiratory questionnaire, SGRQ [11]; chronic respiratory questionnaire, CRQ [12]) and generic health status (medical outcomes short form 36 questionnaire, SF-36 [13]), evaluate both physical and emotional functions and the impact of disease on social function and psychological disturbance [14, 15]. Disease-specific measures have demonstrated greater sensitivity to change from baseline after rehabilitation intervention [16]. However, a significant proportion of eligible patients do not complete the follow-up program and the percentage of patients discontinuing the program in various studies ranges from 20 to 40% [17–20]. Despite the significant percentage of patients discontinuing rehabilitation programs few studies have examined the relevant causes and even fewer studies have focused on psychological factors that differentiate patients who dropped out from those that completed the pulmonary rehabilitation programs. Depression is probably the only psychological factor that has been studied and correlated with dropping out [19]. Depression and depressive symptoms are known to be significantly prevalent in patients with COPD [7, 21–23]. It seems very likely that depressive symptoms are contributing to dropping out. Symptoms include feelings of worthlessness, intense guilt or regret, helplessness or hopelessness, difficulties in concentration and memory, lack of motivation, neglect of personal hygiene, withdrawal from social activities such as family and friendly gatherings, decreased libido, and thoughts of death and suicide [24]. Clinical experience, however, makes us reluctant to fully attribute the phenomenon of dropping out to depressive symptomatology. As a matter of fact, clinicians perceive from the early rehabilitation programs sessions a marked decline in depressive symptomatology that in theory should act in a positive feedback manner by limiting dropout rates. The purpose of this study is to investigate whether there are differences in psychosocial factors among patients with COPD who quit rehabilitation programs and those who complete such programs. We should point out that the study is not intended in any way to exclude patients with COPD from rehabilitation programs due to psychological factors.

## 2. Subjects and Methods

### 2.1. Sample

The study lasted for four years and involved all patients with COPD who presented at a pulmonary rehabilitation program and met the criteria for inclusion in the study. Inclusion criteria in the study were as follows: age less than 80 years without other chronic comorbid conditions (cardiovascular disease, major psychiatric disorders, etc.) and the absence of acute exacerbation of COPD during the last two months before the start of the program. Contraindications included angina, myocardial infarction, severe pulmonary hypertension, congestive heart failure, unstable diabetes, restriction to exercise due to orthopedic or other reasons, dementia (already diagnosed severe cognitive dysfunction or psychiatric illness that interferes with memory and compliance), or severe hypoxia caused by exercise and not corrected by O_2_ administration [25, 26].

### 2.2. Physical Measures

In order to determine COPD severity of our sample, a spirometric evaluation before and after bronchodilation (200 *μ*g salbutamol) was performed. We followed the Global Initiative for Chronic Obstructive Lung Disease (GOLD-updated 2010) diagnostic criteria, which classifies COPD severity (in relation to forced expiratory volume in 1 second (FEV1)/forced vital capacity (FVC) ratio (FEV1%)—percentage of predicted) into four stages: stage I (mild COPD): FEV1 >80% predicted; stage II (moderate COPD): FEV1 50% to 80% of predicted; stage III (severe COPD): FEV1 30% to 50% of predicted; and stage IV (very severe COPD): FEV1 <30% of predicted [27]. The spirometric evaluation of each patient was performed a few days before he/she started the rehabilitation program.

### 2.3. Psychological Measures

The SCL-90-R is a 90-item self-report symptom inventory designed to reflect psychological symptom patterns of psychiatric and medical patients. Each item of the questionnaire is rated on a 5-point scale of distress from 0 (none) to 4 (extreme). The SCL-90-R consists of the following nine primary symptom dimensions: somatization (SOM, which reflects distress arising from bodily perceptions), obsessive-compulsive (OC, which reflects obsessive-compulsive symptoms), interpersonal sensitivity (IS, which reflects feelings of personal inadequacy and inferiority in comparison with others), depression (DEP, which reflects depressive symptoms, as well as lack of motivation), anxiety (ANX, which reflects anxiety symptoms and tension), hostility (HOS, which reflects symptoms of negative reflects, aggression, and irritability), phobic anxiety (PHO, which reflects symptoms of persistent fears as responses to specific conditions), paranoid ideation (PAR, which reflects symptoms of projective thinking, hostility, suspiciousness, and fear of loss of autonomy), and psychotism (PSY, which reflects a broad of symptoms from mild interpersonal alienation to dramatic evidence of psychosis) [28, 29].

The SCL-90 takes between 12 and 20 min to complete. With regard to its reliability, the internal consistency coefficient *α* values for the nine symptom dimensions ranged from 0.77 for psychotism to a high of 0.90 for depression. Additionally, the few validity studies of the SCL-90-R demonstrate that this scale has equal validity compared with other self-report inventories. The SCL-90-R has been standardized and used in the Greek population and its reliability (Cronbach's *α*) for the total of the items is 0.97 [30–32]. The cutoff for the SCL-90-R subscales is 0.99 [32].

The inventory was completed in the presence of psychologists who provided clarifications when necessary.

### 2.4. Pulmonary Rehabilitation Program

Patients of our study followed a pulmonary rehabilitation program for a period of three months, with three sessions per week, each lasting 50 minutes. The program included respiratory physiotherapy, respiratory muscle training, aerobic exercise on a bicycle ergometer and on a treadmill, and strengthening of muscle groups. The exercise was performed with oxygen supplementation while simultaneously recording heart rate and hemoglobin saturation. The minimum and maximum number of sessions per patient was 34 and 39, respectively, with an average of 37 per patient.

Dropping out was predefined as being absent from five consecutive sessions or from 20% of all sessions. In fact all dropout patients were patients fulfilling the first definition of dropping out from the program. Dropout patients were given the chance to start again in a subsequent rehabilitation program.

### 2.5. Statistical Analysis

Statistical analysis was performed using *χ* ^2^ test, paired *t*-test, ANOVA, sample *t*-test, Pearson correlation, and logistic regression. For regression models, an empirical approach was used after correlation analysis. Statistical significance was set at *P* < 0.05 and all the analyses were done with SPSS 19.

The hospital ethics committee approved the study and all participants provided written informed consent. No financial support was necessary.

## 3. Results

The study included 144 patients, 43 (29.9%) of whom did not complete the program and without any manifestation of COPD relapse. One hundred twelve men (77.8%) and 32 women (22.2%) were studied.  Table 1 shows the years of education, FEV1%, disease duration, and stage per GOLD. The sample is not statistically different compared with the general population of patients with COPD in Greece in terms of gender (*χ* ^2^ > 0,05) and age (*t*-test *P* > 0,05) [33]. The female population did not differ from males (*t*-test *P* > 0,05) in disease duration (8,05  ±  9,02 to 8,91  ±  6,01), years of education (11,9  ±  4,02 versus 10,52  ±  4,02), and age (63,65  ±  7,74 to 65,01  ±  8,04, Table 1). Males had lower FV1% compared to females (40,74  ±  20,21 to 52,22  ±  22,43 *t*-test *P* < 0,05, Table 1).

### 3.1. Psychopathology in Patients with COPD

On the SCL-90-R scale 55.6% of patients had abnormal findings (Table 2). High rates were observed for depression (36.1%), somatization (33.3%), compulsion (30.65%), and anxiety (23.7%), while low levels were noted for psychotism (4.9%), phobic anxiety (12.9%), and paranoid ideation (16.7%). Among the 80 patients (55.6%) with positive findings, 60% were positive in more than two scales, while only 23.8% were positive in only one scale of the SCL-90-R. In the SCL-90-R scale patients with very severe COPD showed higher averages in terms of somatization compared to patients with mild COPD (ANOVA test *P* < 0.05) but no statistically significant differences in the other subscales (ANOVA test *P* > 0.05, Table 3).

### 3.2. Dropout Patients

Thirty percent of patients (*N* = 43; 32 men and 11 women) discontinued the program before completion. They did not differ in gender (*χ* ^2^ *P* > 0,05), age (66,05  ±  7,5 years versus 64,15  ±  8,1 years for those who were attentive, *t*-test *P* > 0,05), or disease duration (7,9  ±  8,3 years versus 9,0  ±  6,6 for those who were attentive *t*-test *P* > 0,05) from patients who completed the program. There was no difference in the FV1% (42.6  ±  20,5 for those who discontinued versus 43,5  ±  21,5 of the others, *t*-test *P* > 0,05). Chi-square test revealed no difference concerning disease severity per GOLD criteria (*χ* ^2^ *P* > 0,05). Patients who completed the program had more advanced education (11,3  ±  4,1 versus 9,8  ±  3,8, *t*-test *P* < 0,05). Patients who discontinued the program had higher rates of depression (1,08 versus 0,81, *t*-test *P* < 0,05) and somatization (1,01 versus 0,70, *t*-test *P* < 0,05) compared to those who completed it (Table 4). Regarding the psychopathology scales of SCL-90-R we found that patients who discontinued the program showed higher levels of psychopathology on the scales of somatization, depression, paranoid ideation, and psychotism compared to those who completed it (*χ* ^2^ *P* < 0,05, Table 2).

To determine which variable distinguished better patients who discontinued from patients who completed the program, we performed a binomial logistic regression with years of education and (from the SCL-90-R) whether or not there was psychopathology present (somatization, depression, and paranoid ideation) as covariates. The final regression model showed that people with low educational status and psychoticism were more likely to leave the program. However, the adjustment of the resulting model to the data was not satisfactory (Cox & Snell Pseudo-R2 0,077).

## 4. Discussion

In this study of dropping out from a COPD rehabilitation program, patients who did not complete the program did not differ from those who completed in terms of gender or illness severity. High rates of psychopathology in patients with COPD have been identified in several studies [7, 21–23]. In this study we tried to examine whether this psychopathology contributes to patients dropping out from a COPD rehabilitation program. Dropping out, apart from the financial cost, results in frustration and disappointment to both health professionals and patients. Furthermore it is still unknown what the consequences are for patients remaining in the rehabilitation program. We know that rehabilitation can enhance the psychological aspects of patients who complete the program [7], but we do not know whether this improvement is negatively or positively associated with dropout rates. Our findings point to psychological factors being involved in quitting the rehabilitation program. More specifically, patients who left the program seemed to have higher rates of depression and somatization and among them we found higher rates of pathological psychotic features. We have indications that behind the abandonment of the program it is possible, in terms of psychological parameters, to find psychotic elements. A link with some incipient organic brain syndrome may be possible, since patients of our sample had no history of mental disorder, while the age of these patients made them highly unlikely to show emerging schizophrenia. On the other hand, COPD patients are particularly vulnerable for dementia syndromes [34]. Systemic inflammation is likely to be the common factor linking the two diseases; acute and chronic effects of inflammation in the brain have been associated with cognitive decline and risk of dementia in older adults [34]. Studies show that depressive symptoms are associated with an increase in proinflammatory cytokines and that the level of cytokines corresponds to the severity of depressive symptoms [35, 36]. Depression, in turn, can negatively affect cognitive function by interfering with working memory, executive function, and processing speed. Additionally, depression and depressive symptoms are associated with increased risk of cognitive impairment and dementia among the elderly [37].

Low educational level is a risk factor for dementia syndromes, while a high level of education is considered to be a protective factor [38]. Patients who left the rehabilitation program appeared to feel more physical symptoms compared to those that did not quit; perhaps this is a separate dropout factor. The close relationship between depression and somatization [39] can explain equally well the high percentages of patients who left the program.

It is very likely that the main elements of a pulmonary rehabilitation program that have a positive effect on patients who complete it are the same that make some patients drop out of it. Being a patient in a pulmonary rehabilitation program, mutatis mutandis works in a way analogous to group function [40]; it is formed by people who share common characteristics; it may act therapeutically while it can expel patients with psychotic elements. The sense of belonging to a group is often beneficial: it gives participants the opportunity to interact and through this process to recognize elements of their personal experiences in others as well as to process these elements [41]. However, this is hardly tolerated by some patients. The feeling of the individual that he/she is acceptable, the sense of belonging to a group, the recognition of elements of personal experience in others, identification with others, and emotional contact with other patients and therapists within the program provide help to most COPD patients [7] and may turn away psychotic patients from the program.

We have to point out that the aim of the study was not, in any case, to exclude patients with COPD from the process of rehabilitation. It is very likely that individual rehabilitation programs can help and be well tolerated by COPD patients who for some reason cannot function well within a group. Further research should examine whether there is a direct relationship between cognitive deficits and dropping out from rehabilitation programs.

## 5. Conclusion

Psychological factors in patients with COPD potentially contribute to refraining from participation in pulmonary rehabilitation programs. Psychological evaluation of patients during the selection process for rehabilitation programs may reduce dropout rates.

## Figures and Tables

**Table 1:** Sex, education, FEV1%, and years of diagnosis.

		Age	Education (years)	FEV1%	Years of diagnosis
Male	Mean	65.0179	10.5268	40.7428*	8.9118
	*N* = 112				
	Std. deviation	8.04602	4.02013	20.20831	6.01195

Female	Mean	63.6563	11.9063	52.2203*	8.0588
	*N* = 32				
	Std. deviation	7.74017	4.02700	22.43287	9.28352

Total	Mean	64.7153	10.8333	43.2379	8.6275
	*N* = 144				
	Std. deviation	7.97256	4.04866	21.16720	7.18320

COPD staging per GOLD criteria: mild: *N* = 12, moderate: *N* = 27, severe: *N* = 60, very severe: *N* = 45.

*t-test *P* < 0.05.

**Table 2:** Percentages of pathological values in SCL-90-R.

(*N* = 144)	Total	Male	Female	Dropout	Patients who remained in the program
	(*N* = 144)	(*N* = 112)	(*N* = 32)	(*N* = 43)	(*N* = 101)
Somatization	33.3%	31.3%	40.6%	46.5%*	27.7%*
Obsessive-compulsive	30.6%	26.8%	43.8%	32.6%	29.7%
Interpersonal sensitivity	13.9%	11.6%	21.9%	20.9%	10.9%
Depression	36.1%	30.4%	56.3%	48.8%*	30.7%*
Anxiety	23.7%	18.8%	40.6%	27.9%	21.8%
Hostility	20.8%	17.0%	34.4%	18.6%	21.8%
Phobic anxiety	12.9%	13.4%	9.4%	18.6%	9.9%
Paranoid ideation	16.7%	15.2%	21.9%	27.9%*	11.9%*
Psychoticism	4.9%	3.6%	9.4%	11.6%*	2%*
Without psychopathology	44.4%	50. 0%	25.0%	35.7%	48.5%

*χ ^2^ *P* < 0.05.

**Table 3:** SCL-90-R scores by GOLD stage.

	Mild COPD		Moderate COPD		Severe COPD		Very severe COPD		Total	
	*N* = 12		*N* = 27		*N* = 60		*N* = 45		*N* = 144	
	Mean	SD	Mean	SD	Mean	SD	Mean	SD	Mean	SD
Somatization	1.1908*	0.73960	0.8227	0.57141	0.7530	0.47151	0.6912*	0.52627	0.7849	0.54514
Obsessive-compulsive	1.1167	0.74813	0.7423	0.55941	0.7632	0.50661	0.7209	0.56844	0.7768	0.56340
Interpersonal sensitivity	0.6383	0.61962	0.5142	0.54969	0.4698	0.45054	0.3800	0.48991	0.4649	0.49788
Depression	1.1875	0.78261	0.7092	0.53305	0.9196	0.61554	0.8814	0.65194	0.8914	0.63288
Anxiety	0.8925	0.81761	0.6577	0.58663	0.6228	0.55870	0.6674	0.53529	0.6667	0.58068
Hostility	0.8708	0.79086	0.5062	0.66781	0.5771	0.73508	0.4356	0.53662	0.5450	0.67371
Phobic anxiety	0.4267	0.80009	0.4454	0.79923	0.2984	0.51654	0.4321	0.57746	0.3789	0.61958
Paranoid ideation	0.5383	0.72665	0.6592	0.68492	0.4291	0.48233	0.3658	0.57452	0.4622	0.57925
Psychoticism	0.3000	0.59544	0.2000	0.33226	0.1649	0.27742	0.1442	0.39176	0.1768	0.35909

*ANOVA test *P* < 0.05.

**Table 4:** Means of SCL-90.

	Dropout		Patients who remained in the program	
	*N* = 43		*N* = 101	
	Mean	Std. deviation	Mean	Std. deviation
Somatization	1.0056*	0.65567	0.7012*	0.45826
Obsessive-compulsive	0.8488	0.71759	0.7475	0.47741
Interpersonal sensitivity	0.5281	0.64144	0.4376	0.42644
Depression	1.0791*	0.72996	0.8139*	0.55606
Anxiety	0.7863	0.64028	0.6307	0.56351
Hostility	0.6000	0.78412	0.5340	0.61777
Phobic anxiety	0.4921	0.69419	0.3306	0.56846
Paranoid ideation	0.5616	0.77954	0.4269	0.47995
Psychoticism	0.2674	0.54890	0.1505	0.25598

*t-test *P* < 0.05.
